# Reflective Quasi-Continuous Metasurface with Continuous Phase Control for Light Focusing

**DOI:** 10.3390/ma14092147

**Published:** 2021-04-23

**Authors:** Long Chen, Zhenglong Shao, Jia Liu, Dongliang Tang

**Affiliations:** Key Laboratory for Micro/Nano Optoelectronic Devices of Ministry of Education & Hunan Provincial Key Laboratory of Low-Dimensional Structural Physics and Devices, School of Physics and Electronics, Hunan University, Changsha 410082, China; longchen20@hnu.edu.cn (L.C.); zlshao@hnu.edu.cn (Z.S.); jialiu@hnu.edu.cn (J.L.)

**Keywords:** quasi-continuous, metasurface, all-metallic, diffraction limit

## Abstract

Benefitting from the arbitrary and flexible light modulation, metasurface has attracted extensive attention and been demonstrated in different applications. However, most reported metasurface-based devices were normally composed of discrete micro or nano structures, therefore the discretization precision limited the performance of the device, including the focusing efficiency, stray light suppression, and broadband performance. In this work, an all-metallic reflective metasurface consisting of numerous quasi-continuous nanostructures is proposed to realize high-efficiency and broadband focusing. The constructed quasi-continuous metasurface (QCMS) is then verified numerically through electromagnetic simulation, and the numerical results show a higher focusing efficiency and a better stray light suppression effect, compared to a binary-phase-based metalens. Through the same design strategy, a QCMS with the ability to overcome the diffraction limit can also be constructed, and a focal spot with the size of 0.8 times the diffraction limit can be achieved. We expect that this quasi-continuous structure could be utilized to construct other high-performance devices that require continuous phase controls, such as the beam deflector, orbital angle momentum generator, and self-accelerating beam generator.

## 1. Introduction

Phase not only plays an important role in physics, but also in mathematics. It is an essential term to describe the electromagnetic characteristics of light. A variety of applications can be realized by the accurate phase control, such as a focusing lens [1], beam deflector [2] and vortex beam generator [3]. Traditional phase control is based on the suitable geometric shape of the medium so that electromagnetic waves can achieve a required phase delay. This common method is extensively applied to design various optical devices, such as the optical lens [4], quarter-wave plate [5] and spiral phase plate [6]. However, the refractive index of the natural material is typically small in the visible spectrum, thereby the conventional optical element requires a large thickness compared to the operating wavelength for the sufficient phase retardation. Such shape and thickness significantly limit the miniaturization and integration of the device and system.

In recent years, an attractive artificial two-dimensional (2D) metamaterial, also known as metasurface, has been demonstrated to control the wavefront flexibly at subwavelength resolution. It is regarded as an ideal candidate to construct a miniaturized and compact optical element. The metasurface controls phase, amplitude, and even polarization of electromagnetic wave by changing the geometric parameters such as the length, width and rotation angle of micro/nano structure [7,8,9]. In addition, using a metamaterial loaded with a liquid crystal can also achieve the control of electromagnetic wave [10]. These are entirely different modulations compared with the conventional propagation phase. Based on the flexible wavefront modulation, metasurface has been successfully employed to realize a series of applications, such as the light focusing [7,8,9], optical hologram [11,12,13] and anti-counterfeit technology [14,15]. Among these reported metasurfaces, the metasurface based on the Pancharatnam Berry (PB) phase [7,16] (also named the geometric phase) was widely exploited owing to its simple phase control which is only related to the rotation angle of the unit structure. Although this kind of metasurface can provide dispersionless phase shift with a simple design process, there are still some problems that should be solved in practical applications. For instance, most reported metasurfaces were composed of discrete micro/nano structures [15,16], and each individual structure only introduced a local phase and amplitude. It means that the desired continuous phase/amplitude profile of light wave must be discretized. Such a discrete design inevitably reduces the energy efficiency and signalnoise ratio. In addition, the discrete metasurface possessed a relatively high diffraction efficiency only in the special spectral region due to the electromagnetic resonance [17], which limited the working bandwidth of the device. To address the mentioned drawback, a quasi-continuous structure, called the catenary [17,18,19,20,21,22], has been proposed in recent years. The catenary can continuously control the phase from 0 to 2π and generate the wavelength-independent phase shift as it is derived from the PB phase. Its theoretical energy efficiency does not change with different incident light wavelengths in the visible spectrum, particularly when the characteristic dimension of catenary is at deep sub-wavelength magnitude. This quasi-continuous metasurface has been widely used to design the vortex beam generator [18], deflector [20] and holography [23,24]. However, most of the reported quasi-continuous metasurfaces were composed of plasmonic structures [20,23], and the transmissions of such nano-structures were limited.

In this work, an all-metallic quasi-continuous metasurface (QCMS), consisting of continuous space-variant equidistant catenary-shaped aluminum nanostructures, is proposed for high-efficiency and broadband light focusing. Instead of periodically changing the rotation angle of nanostructures, the phase gradient covering from 0 to 2π can be achieved by a single catenary-shaped nanostructure. Based on this nanostructure, we numerically design two optical QCMSs for diffraction-limited focusing and sub-diffraction focusing under circular polarized light incidence, respectively. Through the electromagnetic simulation, the former generates the focusing spot with almost diffraction-limited spot size, while the latter forms that with 0.8 times the Airy spot. The operating bandwidth of our QCMS covers the visible spectrum from 450 nm to 650 nm, and the focusing efficiency is several times than the binary-phase-based metalens. Simulated results agree well with theoretical analyses. This catenary-shaped nanostructure can be also scaled to the infrared, terahertz and microwave wavelength without complicated optimization. We expect that it can provide a valid platform for the realization of various high-efficiency electromagnetic components.

## 2. Principles

**Figure 1:**
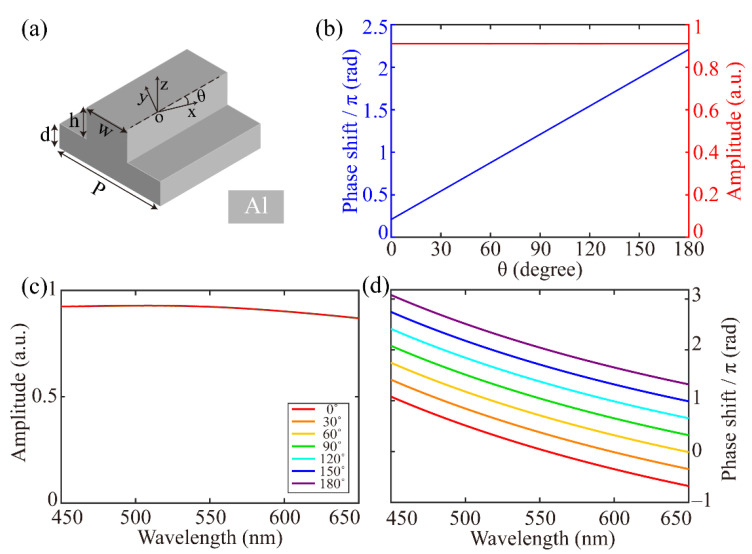
(**a**) Simplified model of a catenary-shaped nanostructure. (**b**) Simulated phase (blue line) and amplitude (red line) profiles of reflective cross-polarization light at the wavelength of 532 nm. Simulated amplitude (**c**) and phase (**d**) profiles for different wavelengths and rotation angles.

Our proposed single catenary-shaped nanostructure can be discretized into numerous subunits, which are used as the basis models for the analysis of light modulation. The schematic diagram of the subunit is shown in Figure 1a, where the aluminum (Al) nanobrick with the height (*h*) and width (*w*) is deposited on the Al substrate with the thickness (*d*) and period (*P*), and the rotation angle (*θ*) is defined as the angle between the long-axis of nanobrick and the x-axis. Benefitting from the physical properties (such as malleability, high strength, and heat resistance) of the metal, the all-metallic structures used are more significant and easy to fabricate in practical applications than all-dielectric and plasmonic structures [16]. To accurately calculate optical performances of the subunit, the *h*, *w*, *d* and *P* are fixed at 150, 60, 110 and 300 nm (the values of these parameters are optimized for the purpose of obtaining the highest reflectance in the visible spectrum), respectively, and the *θ* is swept from 0 degree to 180 degree in steps of 30 degree in CST microwave studio software. As the left circular polarized (LCP) light illuminates the subunit, the reflective phase (blue line) and amplitude (red line) are depicted in Figure 1b, and it is clearly shown in the figure that there is a linear relation of *φ* = 2*θ* between the phase shift and rotation angle. The calculation indicates the PB phase principle and agrees well with it. Furthermore, the reflective amplitudes are almost above 0.9, and do not change with the rotation angle. The relations between the amplitude and wavelength at different rotation angle are depicted in Figure 1c, which proves that the subunit possesses the broadband amplitude. It can provide dispersionless phase shifts as illustrated in Figure 1d. The above superior properties of the subunit indicate that the catenary-shaped nanostructure is an ideal candidate for constructing high-efficiency broadband optical devices.

**Figure 2:**
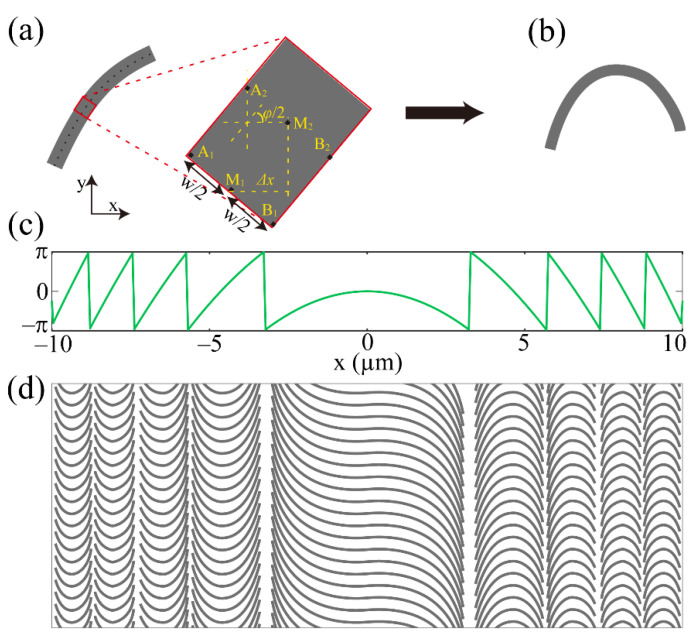
(**a**,**b**) Schematic diagram of the design process. (**c**) Ideal phase profiles of the QCMS. (**d**) Constructed QCMS with an area of 20 μm × 10 μm.

Based on the above simplified model and numerical calculations, we first design a cylindrical lens flat lens. The required phase profile can be expressed as [20]:

(1) $$\varphi \left(x\right)=\frac{2\pi }{\lambda }\left(f-\sqrt{x^{2}+f^{2}}\right)$$

where *λ* and *f* are the working wavelength and focal length, respectively. The lens construction can be divided into the design of catenary-shaped nanostructures along x-axis and the periodic arrangement along y-axis. In the Cartesian coordinate system, the center line of a catenary-shaped nanostructure can be discretized into N points (M_1_, M_N_) with equal spacing of Δ*x* along the x-axis, as shown in Figure 2a. The rectangle in the red box is an enlarged view of a part of the nanostructure, and points A_1_, B_1_, A_2_, B_2_, A_N_, and B_N_ are located at the boundary of the nanostructure. These points are centrosymmetric about points M_1_, M_2_, and M_N_, respectively, and A_1_B_1_, A_2_B_2_, and A_N_B_N_, respectively, represent the distance or width between A_1_ and B_1_, A_2_ and B_2_, and A_N_ and B_N_. If the starting point is M_1_(*x*_1_, *y*_1_) with the rotation angle of *φ*(*x*_1_)/2, then the coordinates of points A_1_(*x_A_*_1_, *y_A_*_1_) and B_1_(*x_B_*_1_, *y_B_*_1_) can be depicted as: *x_A_*_1_ = *x*_1_ – *w*/2 sin(*φ*(*x*_1_)/2), *y_A_*_1_ = *y*_1_ + *w*/2 cos(*φ*(*x*_1_)/2), *x_B_*_1_ = *x*_1_ + *w*/2 sin(*φ*(*x*_1_)/2), *y_B_*_1_ = *y*_1_ – *w*/2 cos(*φ*(*x*_1_)/2), and the coordinates of the next point M_2_(*x*_2_, *y*_2_) can be calculated: *x*_2_ = *x*_1_ + *Δx*, *y*_2_ = *y*_1_ + *Δx* tan(*φ*(*x*_2_)/2). According to the similar mathematical law, we can calculate the coordinates of A_2_, A_N_, and B_2_, B_N_, and then connect them to draw up the catenary-shaped nanostructure, as shown in Figure 2b. Based on the ideal phase profiles shown in Figure 2c, the nanostructures along x-axis are built through above design processes (*Δx* is set as 30 nm), and then the QCMS can be obtained by periodically arranging these nanostructures along the y-axis, and the schematic diagram is depicted in Figure 2d where the interval between two adjacent catenary-shaped nanostructures along the y-axis is 300 nm. It is worth pointing out that the structure should be truncated at some positions along the x-axis because the value of tan(*φ*(*x*)/2) at *φ* = ±π is infinite.

## 3. Results and Discussion

**Figure 3:**
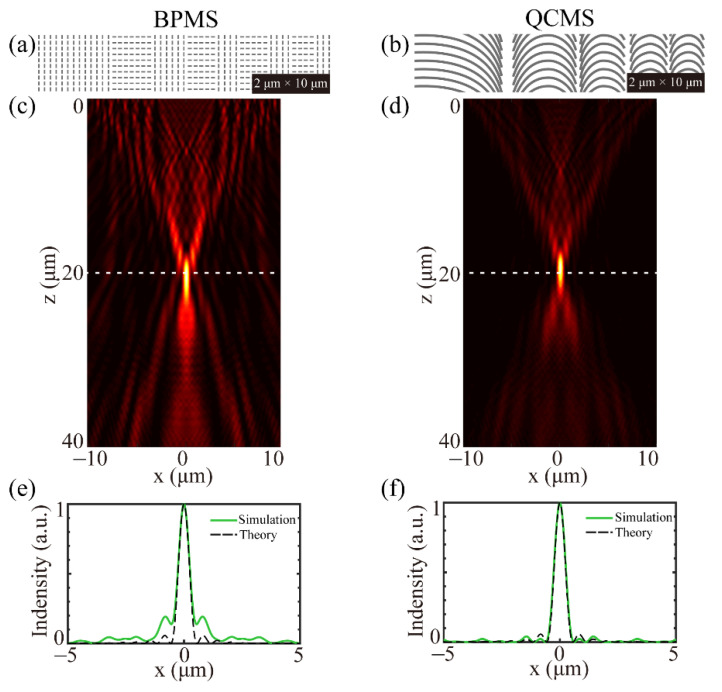
(**a**,**b**) Partial diagrams of BPMS and QCMS, respectively. Light fields of BPMS (**c**) and QCMS (**d**) along the propagating direction. (**e**,**f**) Corresponding normalized intensity curves along the white dotted lines in (**c**,**d**), where black dotted lines indicate the theory intensity curves.

To verify the availability of our proposed method, a binary phase-type metasurface (BPMS) with the same parameters can be constructed, as presented in Figure 3a, where there are only two orientations of 0 and 90 degrees. The focusing performances of these constructed metasurfaces are evaluated by the finite-difference time-domain method in CST software, where the incident light is assumed to be LCP with the wavelength of 532 nm and the propagating direction along the z-axis. The lower and upper boundary from the metasurface in CST are 50 nm and 500 nm, respectively, and the open boundary conditions are used along the x-axis, y-axis, and z-axis. The total mesh cells in the calculation range are about 3.3 × 10^7^ to ensure there are at least two mesh cells in the minimum nanostructure. To effectively reduce the computation time, the reflective field distribution at a distance of 100 nm from the structure surface is extracted and then used to calculated the follow-up propagation of light based on scalar angular spectrum theory (SAS) [25,26]. SAS is a calculation method using the angular spectrum of the plane wave to calculate the field distribution at arbitrary position along the propagating direction. This method ignores the longitudinal electrical component of far-field in the calculation. If the longitudinal component is too large, such as in a high-NA system, SAS is no longer applicable. The simulated axial light fields of BPMS and QCMS are illustrated in Figure 3c,d, respectively, where the white dotted lines indicate the focal plane. The corresponding normalized intensity curves along the x-axis in Figure 3c,d are depicted in Figure 3e,f, where the black dotted lines indicate the results calculated by SAS. It is clearly seen that the undesired stray lights are highly suppressed through a continuous phase control. In addition, the focusing efficiency, defined as the ratio of the energy concentrated into the hotspot to the incident energy, is 42.48% for QCMS, while it is 29.22% for BPMS. If the incident intensity is assumed to be 1, the focal intensities are 16.21 for QCMS and 9.72 for BPMS. The focusing performance proves the superiority of catenary-shaped nanostructures. Although we only present the focusing performances of QCMS and BPMS, our proposed QCMS can show a better suppression of the undesired stray lights through comparing it to metalens with a four-level phase.

**Figure 4:**
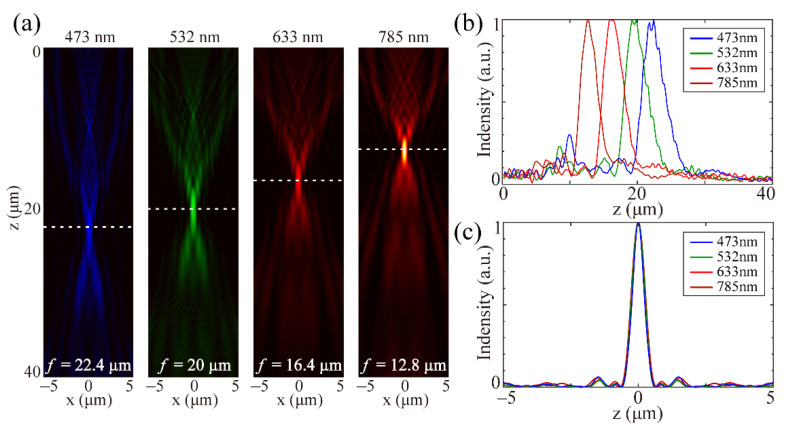
(**a**) Axial light field distributions at the wavelengths of 473, 532, 633, and 785 nm. (**b**) Normalized intensity curves along the propagating direction. (**c**) Corresponding normalized intensity curves along the white dotted lines in (**a**).

As the phase control of the metasurface comes from the PB phase, the device provides the same phase shift at different wavelengths. According to Equation (1), when *φ* is a constant, *f* is nearly inversely proportional to *λ*. Therefore, there are axial chromatic aberrations at different incident wavelengths. We have calculated the reflective light distributions at the wavelengths of 473, 633 and 785 nm, respectively, as shown in Figure 4. The light distributions along the optical axis for the three wavelengths are almost the same as that for 532 nm, except for the large chromatic focus shift in Figure 4a. The results are consistent with previous reported references where the focal length changes inversely with the wavelengths [7]. The full width at half-maximums (FWHMs) of the focal spots are 1.025, 1.080, 1.073 and 1.098 μm at the wavelength of 473, 532, 633 and 785 nm, respectively, which presents a nearly unchanging diffraction pattern, as shown in Figure 4c. This invariance of focal spots is derived from the chromatic focus shift defined by constant *λz* under the paraxial approximation [7]. The detailed optical performances of the QCMS are presented in Table 1, where the incident intensity is assumed to be 1.

**Figure 5:**
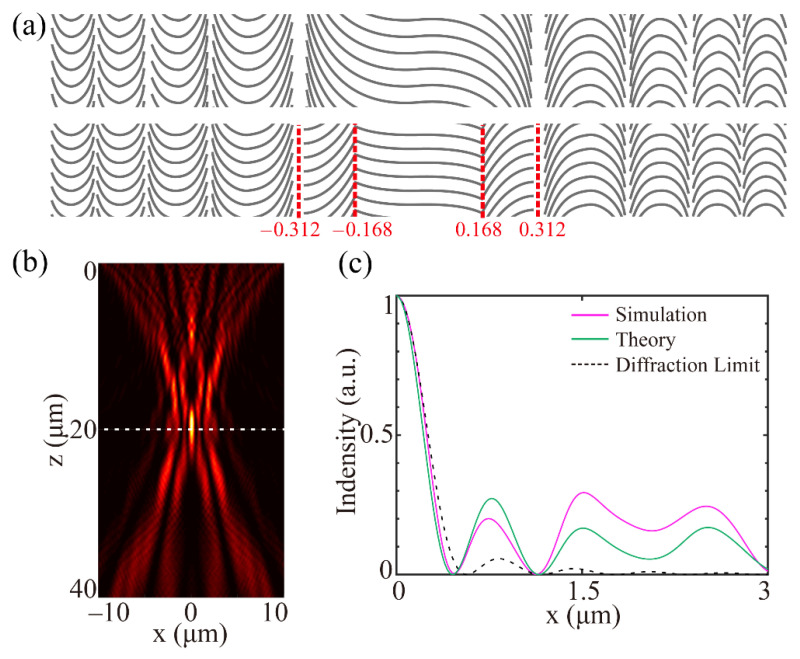
(**a**) Schematic of the QCMS (top row) and SOQCMS (bottom row), the red numbers indicate the normalized π-phase-jump radial positions. (**b**) Simulated field distribution along the propagating direction at the wavelength of 532 nm. (**c**) Normalized intensity curve along the white line in (**b**).

To further verify the flexibility of the proposed method, we design a super-oscillation QCMS (SOQCMS) for sub-diffraction focusing on the wavelength of 532 nm with the dimensions of 20 μm × 10 μm and the focal length of 20 μm. According to our previous works [7,27], an extra binary super-oscillation phase can be added to the phase profile of a lens to realize sub-diffraction light modulation. Based on the required parameters of the sub-diffraction focal spot, the binary super-oscillation phase can be optimized reversely by the linear programming method (LPM), particle swarm optimization (PSO) or genetic algorithm (GA) [26,27]. In this design, the radius at first zero intensity of the super-oscillatory spot is set as 0.8 times that of the diffraction-limited spot, and the ratio of the maximum side-lobe intensity to the central intensity, i.e., the M value, is set to 0.25. After sufficient iterations, the normalized π-phase-jump radial positions are calculated at 0.168 and 0.312. Thus, the ideal phase profile of the SOQCMS can be described as *φ_sol_* = *φ*(*x*) + *φ_binary_*, where *φ*(*x*) indicates the phase profile of the aforementioned flat lens, and *φ_binary_* indicates the binary super-oscillation phase. By using the design process proposed above, the SOQCMS is constructed, and the schematic diagram is depicted at the bottom of Figure 5a, where the red dotted lines represent the π-phase-jump positions. Through the electromagnetic simulation, the intensity distribution along the optical axis is shown in Figure 5b, and the normalized transverse curve at focal plane (z = 20 μm) is illustrated in Figure 5c, where the green line presents the results calculated by SAS, and black dotted line indicates the intensity profiles of Airy spot. The radius at first zero intensity of the super-oscillatory spot is 470.6 nm, 0.803 times that of the diffraction-limited radius, and the M value is 0.294. There are some deviations between simulated and theoretical results, which originate from the following reasons: First, some slight differences between the achieved and ideal phase profiles; second, the coupling between adjacent quasi-continuous structures may cause some undesired phase shifts. Compared with multi-wavelength achromatic super-oscillation metasurfaces [28], the correction of the axial aberration is not considered in the optimization of our devices. Thereby it is difficult to achieve achromatic performance through our quasi-continuous PB-based metasurface. A potential solution is merging PB phase and transmission phase reported in Ref. [29].

**Figure 6:**
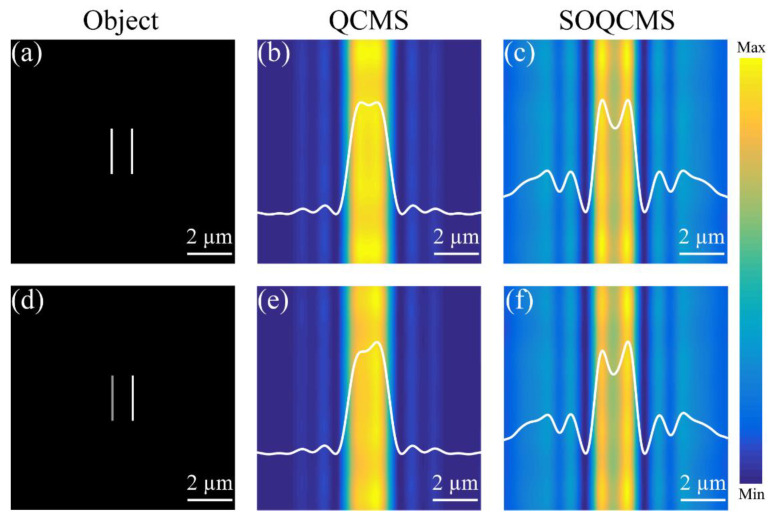
Simulated imaging results of two transparent equidistant slits with the same grayscales (**a**) and different grayscales (**d**) by employed QCMS (**b**,**e**) and SOQCMS (**c**,**f**), respectively. The white lines present the corresponding intensity curves. Both devices are illuminated under LCP light at the wavelength of 532 nm.

Next, the super-resolution imaging performance of our SOQCMS is demonstrated. We assume that the object is set at the front focal plane of a lens that has the same focal length as our SOQCMS. Then, our devices are placed behind the lens to collect the transmitted parallel light for imaging. For an arbitrary object, the corresponding image is the two-dimensional convolution between the focal spot and the object. We choose two slits in Figure 6a,d as the object. The center-to-center distance of slits are 856 nm (about 0.73 times the diffraction-limited distance), and the ratio of grayscales between two slits are 1:1 in Figure 6a and 0.9:1 in Figure 6d. The imaging results are shown in Figure 6, where the white lines in Figure 6 indicate the normalized intensity profiles. Obviously, the selected object cannot be observed with the QCMS, while it can be easily distinguished by using the SOQCMS. Some speckles on the image plane are caused by sidelobes surrounding the central focal spot. In actual applications, SOQCMS can be integrated into a confocal scanning microscopy for imaging [30] or for the in-field-of-view imaging [31].

## 4. Conclusions

In summary, a high-performance metasurface with continuous wavefront manipulation is proposed and constructed through an all-metallic, equidistant and catenary-shaped nanostructure. The simplified nanostructure and its electromagnetic responses are numerically analyzed. Then, we construct the corresponding QCMS and verify the better focusing performance compared with the binary-phase-based metalens. The broadband property is also demonstrated because of the dispersionless phase shift provided. In addition, the design strategy is employed to design a super-oscillatory metalens for sub-diffraction focusing and super-resolution imaging. The all-metallic structures may afford new ideas for the research in interdisciplinary fields owing to the electrical and thermal conductivity of the metal, and the proposed method could be employed in various actual engineering such as the OAM generation, beam steering, and self-accelerating beam generator.

## Figures and Tables

**Table 1:** Optical performance of the QCMS.

**Wavelength (nm)**	473	532	633	785
**Focal Length (μm)**	22.4	20	16.4	12.8
**Central Intensity**	15.89	16.21	19.28	16.71
**FWHM (μm)**	1.025	1.08	1.073	1.098
**Focusing Efficiency (%)**	40.76	42.48	52.62	48.37

## Data Availability

Data sharing not applicable.
